# The Changes in the Antibiotic Resistance of *Staphylococcus aureus*, *Streptococcus pneumoniae*, *Enterococcus faecalis* and *Enterococcus faecium* in the Clinical Isolates of a Multiprofile Hospital over 6 Years (2017–2022)

**DOI:** 10.3390/jcm14020332

**Published:** 2025-01-08

**Authors:** Agnieszka Jama-Kmiecik, Beata Mączyńska, Magdalena Frej-Mądrzak, Irena Choroszy-Król, Ruth Dudek-Wicher, Daniel Piątek, Krzysztof Kujawa, Jolanta Sarowska

**Affiliations:** 1 Department of Medical Biology, Faculty of Nursing and Midwifery, Wroclaw Medical University, 50-368 Wroclaw, Poland; agnieszka.jama-kmiecik@umw.edu.pl (A.J.-K.); irena.choroszy-krol@umw.edu.pl (I.C.-K.); jolanta.sarowska@umw.edu.pl (J.S.); 2 Department of Hygiene and Epidemiology, Lower Silesian T. Marciniak Specialist Hospital-Center for Emergency Medicine, 54-049 Wroclaw, Poland; beata.maczynska@umw.edu.pl; 3 Department of Pharmaceutical Microbiology and Parasitology, Faculty of Pharmacy, Medical University, 50-367 Wroclaw, Poland; ruth.dudek-wicher@umw.edu.pl; 4 Department of Microbiology, Faculty of Medicine, Medical University, 50-367 Wroclaw, Poland; 5 Lower Silesian T. Marciniak Specialist Hospital-Center for Emergency Medicine, 54-049 Wroclaw, Poland; danielpiatek222@wp.pl; 6 Statistical Analysis Centre, Medical University, 50-367 Wroclaw, Poland; krzysztof.kujawa@umw.edu.pl

**Keywords:** multidrug-resistant strains, Gram-positive coccus, fluoroquinolones

## Abstract

**Background/Objectives:** The growing resistance of bacteria to antibiotics is a serious problem in health care. The present study aims to assess the drug resistance of *Staphylococcus aureus*, *Enterococcus faecium*, *Enterococcus faecalis* and *Streptococcus pneumoniae* isolated from infections in a multispecialty hospital over a 6-year period. **Methods:** Identification and antimicrobial susceptibility testing were performed using the VITEK^®^2 automated system (Biomerieux). **Results and Conclusions:** Based on data from the analyzed hospital, MRSA strains were the etiological factor of 18–28% of *S. aureus* infections. In each year from 2017 to 2022, the percentage of MSSA strains steadily exceeded the number of MRSA strains. The MRSA strains isolated show significant sensitivity to antibiotic groups other than β-lactams, such as aminoglycosides, tetracyclines, cotrimoxazole, linezolid and vancomycin. Ciprofloxacin is the antibiotic to which *S. aureus* displays the second-highest resistance, after methicillin. In the case of MRSA strains, almost 100% lack of sensitivity to quinolines was found. An increase in the number of infections caused by strains of the *Enterococcus* genus was observed. For *E. faecium* strains, the percentage of vancomycin-resistant strains reached as much as 41% in 2018. Among the resistant strains in *E. faecalis*, VREs (Vancomycin-Resistant Enterococci) slightly predominate, while GREs (Glycopeptide-Resistant Enterococci) are much more prevalent in *E. faecium*. The data show that the percentage of *S. pneumoniae* strains insensitive to ampicillin ranged from 6% to 17%. In 2017, the percentage of strains resistant to this antibiotic reached 17%, while in 2022, their share decreased to 9%. In 2021–2022, the percentage of strains resistant to erythromycin was as high as 33%. This resistance is related to the MLS (macrolides, lincosamides, streptogramines B) mechanism. An increase in *S. pneumoniae* resistance to 100% was observed in 2017 and 2019. In the analyzed six-year period, from 2020 (beginning of the pandemic), in some groups of antibiotics, a significant increase in consumption in DDD/100 person-days was recorded. This is most visible in the case of fluoroquinolones. The analysis carried out will increase the effectiveness of empirical therapy in the hospital and the prudent use of antibiotics to limit the selection of multidrug-resistant strains.

## 1. Introduction

The increasing number of antibiotic-resistant microorganism strains has made the treatment of bacterial infections a major problem of modern medicine. The spread of antibiotic-resistant microorganisms is caused by the excessive and inappropriate use of antibiotics, not only in the treatment of human infections but also in veterinary medicine, agriculture and the food industry. Antibiotic treatment options for infections caused by certain pathogens are now limited to a few drugs, and in some cases even to one [1,2]. The rapid increase in antibiotic resistance among Gram-positive cocci is a particularly disturbing phenomenon. A large proportion of the microorganisms analyzed in this study are on the WHO’s priority list of microorganisms. Like other bacteria, over the course of evolution, *Staphylococcus aureus* has developed many antibiotic resistance mechanisms. Among the most clinically important is its lack of sensitivity to β-lactam antibiotics, as β-lactams are the drugs of choice in the treatment of staphylococcal infections [3,4]. Initially, *S. aureus* developed resistance to natural penicillins. Another serious problem is the growing methicillin resistance of *S. aureus*, i.e., its resistance to all β-lactam antibiotics except for fifth-generation cephalosporins—ceftaroline and ceftobiprole [3,5]. The most common mechanism of *Staphylococcus aureus* resistance to aminoglycosides involves their enzymatic modification using acetyltransferase, phosphotransferase and nucleotidyltransferase. The second mechanism responsible for reduced sensitivity to aminoglycosides is the methylation of the receptor for these drugs on the 30S ribosomal subunit [6]. These cocci may also develop the MLS_B_ mechanism, i.e., resistance to macrolides, lincosamides and streptogramins B. These drugs are an alternative for patients allergic to penicillin but also for those infected with MRSA (methicillin-resistant *Staphylococcus aureus*), and the development of resistance to these antibiotics leaves us with one less therapeutic option [7]. Fluoroquinolones are another group of antibiotics to which *S. aureus* may develop resistance [6]. The first *S. aureus* strain showing lack of sensitivity to vancomycin was isolated almost 20 years ago. Today, however, it is known that GRSA (glycopeptide-resistant *S. aureus*) strains can be classified into two groups—those with the *vanA* operon and those without it [8]. The main mechanism of glycopeptide resistance relies on the ability to synthesize an altered cell wall precursor (D-Ala-D-Lac), which results in the inability of the antibiotic to bind to the target site [6].

In enterococci, acquired resistance may be related to a ribosomal mutation on the 30S subunit or it may involve the modification of the antibiotic with the help of an enzyme. This is possible owing to enzymes that modify aminoglycosides (AMEs). As a result of their action, the drug is unable to bind to the target site (the 30S ribosomal subunit) [9,10]. Three phenotypes have been distinguished among strains showing resistance to high concentrations of aminoglycosides: HLGR—showing high resistance to gentamicin; HLSR—showing high resistance to streptomycin; and the phenotype of resistance to high concentrations of kanamycin [10]. *Enterococcus faecium* is one of the most important pathogens in the epidemiology of VRE (Vancomycin-Resistant *Enterococcus*) infections [9,10,11].

The mechanism of resistance to linezolid in LREs (linezoid-resistant enterococci) involves a mutation of the gene encoding 23S rRNA, which prevents the synthesis of bacterial proteins. It occurs relatively rarely but always in *E. faecium* [10]. Additionally, *Enterococcus* spp. can develop resistance to β-lactams [9]. Some of the isolated strains also show resistance to tetracyclines [9,12]. The MLS_B_ (macrolides, lincosamides, streptogramines B) mechanism is also important, in which a mutation leading to structural changes within the ribosome (preventing the antibiotic from binding to it) leads to resistance to all macrolides, lincosamides and streptogramins B [9].

Penicillin was initially the drug of choice for infections caused by *Streptococcus pneumoniae*. The situation changed with the acquisition of the *pbp* resistance gene, which led to the production of modified PBP proteins. They have a reduced affinity for drugs and thus enable the bacteria to synthesize the cell wall even in the presence of an antibiotic [13,14]. *Streptococcus pneumoniae* resistance to macrolides reduces the available therapeutic options for respiratory infections. The above-mentioned bacteria have also developed resistance to fluoroquinolones [15].

Our work aims to assess the drug resistance of *S. aureus*, *E. faecium*, *E. faecalis* and *S. pneumoniae* isolated from infections in a multispecialty hospital over a 6-year period.

## 2. Materials and Methods

### 2.1. Bacterial Strains

The analyzed data came from the resources and materials of the Department of Hygiene and Epidemiology of the Multi-Clinic Specialist Hospital in Wroclaw and cover the period from 1 January 2017 to 31 December 2022. The clinical samples were obtained from hospital patients as part of routine microbiological tests outsourced to the Synevo medical laboratory located on the hospital’s premises. Analyzed microorganisms were isolated in hospital infections mainly from urine, blood, respiratory tract, purulent secretions and other clinical materials. Clinical sample collection was performed according to microbiological standards. The development of antibiotic resistance of the following bacteria was analyzed: *S. aureus* (MRSA and MSSA (*methicillin-susceptible Staphylococcus aureus*) strains), *E. faecalis*, *E. faecium* and *S. pneumoniae* (Table 1).

Resistance to specifically selected antibiotics was analyzed depending on the strain (Table 2).

This study also covered the consumption of antibiotics used to treat Gram-positive bacterial infections in hospitals (in DDD/100 person-days) in the period 2017–2022.

In order to identify microorganisms in the Synevo laboratory, strains were isolated from clinical samples on the appropriate microbiological media. Microbial identification and antimicrobial susceptibility (including MRSA) testing were performed using the automated VITEK^®^2 system (Biomerieux, Craponne, France). The tests used the automated method in the Vitek system based on microdilution in a liquid medium with the determination of the MIC value. Suitable VITEK^®^2 identification cards (GP, AST-P643, AST-P644, AST-ST03) for Gram-positive bacteria were used. These cards also contain specific sets of antibiotics. The results of sensitivity to selected antibiotics important in the treatment of the described microorganisms were analyzed. The susceptibility profile was interpreted according to the criteria of the European Committee on Antimicrobial Susceptibility Testing (EUCAST) for each microorganism analyzed. The Synevo laboratory is not part of the analyzed hospital. However, it is an accredited company with an ISO 2018 certificate and works according to standards using reference strains appropriate to the methods.

### 2.2. Data Analysis

Data illustrating the changes in the antibiotic resistance of the selected bacteria in the years 2017–2022 were analyzed and are presented using Excel charts. The data on antibiotic consumption in the hospital are presented in the same way.

The statistical significance of trends in the resistance of the tested microorganisms to the drugs used in the years 2017–2022 was tested using the Cochran–Armitage test [16]. This test has more power compared to the Chi-square test if a trend is expected. The results of this test are presented using the test value (Z) and *p*-value. Since the Cochran–Armitage test is assigned to 2 × N tables, in the case of *S. pneumoniae* resistance analysis, which analyzed the percentage share of three strains (susceptible, moderately susceptible and resistant), the trends for moderately susceptible and resistant strains were analyzed separately.

## 3. Results

### 3.1. Evolution of Bacterial Resistance

#### 3.1.1. Antibiotic Resistance of *Staphylococcus aureus* Strains in 2017–2022

For the purposes of the analysis of the changes in *S. aureus* resistance over the past 6 years, antibiotic groups such as aminoglycosides, fluoroquinolones, macrolides, lincosamides, cotrimoxazole and rifampicin were considered. However, among the strains isolated from hospital patients, there were no strains resistant to glycopeptides or linezolid. In addition, antibiotic susceptibility was considered separately for MRSA and MSSA.

The average number of MRSA cases was 102.5 and that of MSSA cases was 343.8 over the time period analyzed. The highest number of *S. aureus* infections was in 2018 (n = 627), after which the number of both MRSA and MSSA strains declined and remained at a similar low level. The number of MSSA strains was 406, 450, 364, 287, 267 and 289 and that of MRSA strains was 126, 177, 81, 78, 81 and 72 between 2017 and 2022, respectively.

**Figure 1:**
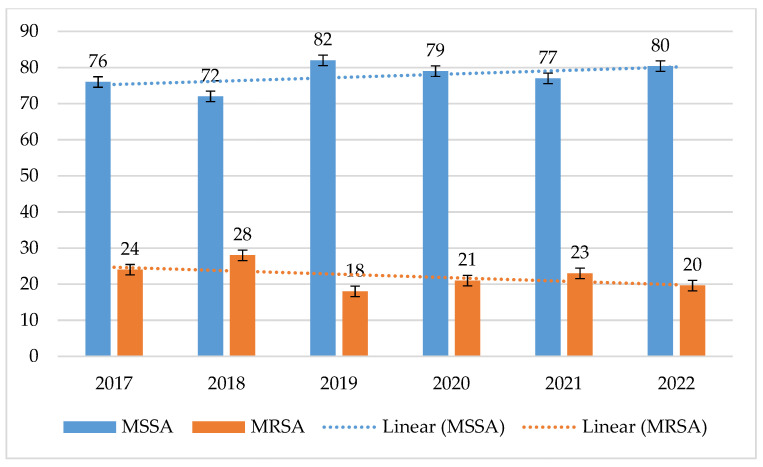
Comparison of the percentage of MSSA and MRSA strains between 2017 and 2022.

In each year of the six-year period from 2017 to 2022, the percentage of MSSA strains steadily exceeded the number of MRSA strains. This percentage ranged from 72% in 2018 to 82% in 2019. In contrast, the percentage of MRSA strains was at a constant low level, ranging from 18% to 28% (Figure 1).

**Figure 2:**
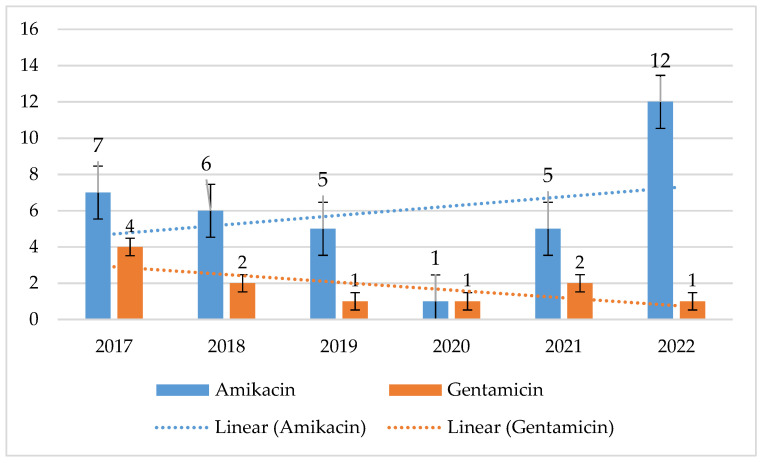
Percentage of MSSA strains resistant to aminoglycosides.

**Figure 3:**
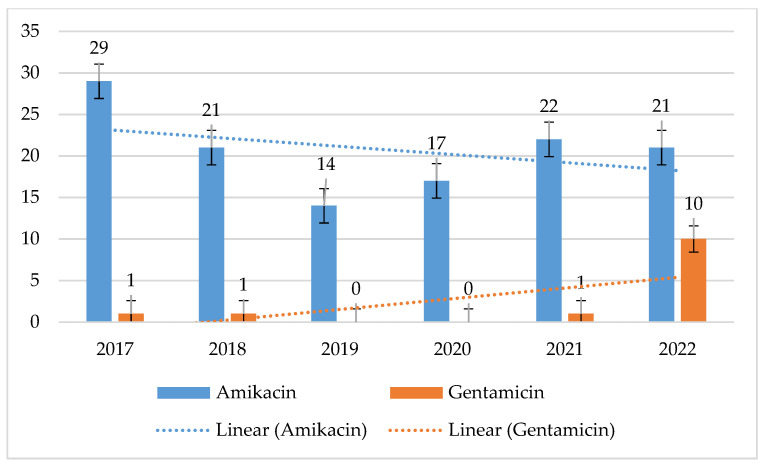
Percentage of aminoglycoside-resistant MRSA strains.

MRSA strains exhibited more than 3 times higher resistance to amikacin than MSSA strains. For MSSA, resistance was low, ranging from 1% to 12% (*p* = 0.1657) with a noticeable upward trend. In contrast, for MRSA, amikacin resistance ranged from 14% to 29% (*p* = 0.1815), with a slightly decreasing trend. However, it should be noted that this percentage was also relatively low for MRSA. The lowest percentage of resistant strains was recorded for MRSA in 2019 and for MSSA in 2022. For gentamicin, both MSSA (*p* = 0.01033) and MRSA (*p* = 0.00078) retained nearly 90% sensitivity. MSSA strains showed a downward trend in gentamicin resistance, while MRSA showed an insignificant increase in 2022 (Figure 2 and Figure 3).

**Figure 4:**
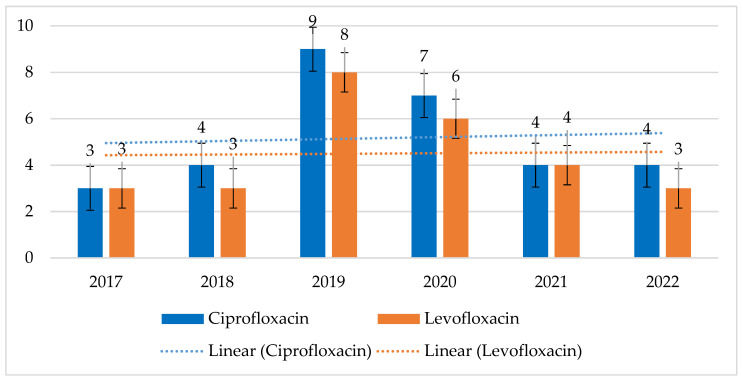
Percentage of MSSA strains resistant to fluoroquinolones.

**Figure 5:**
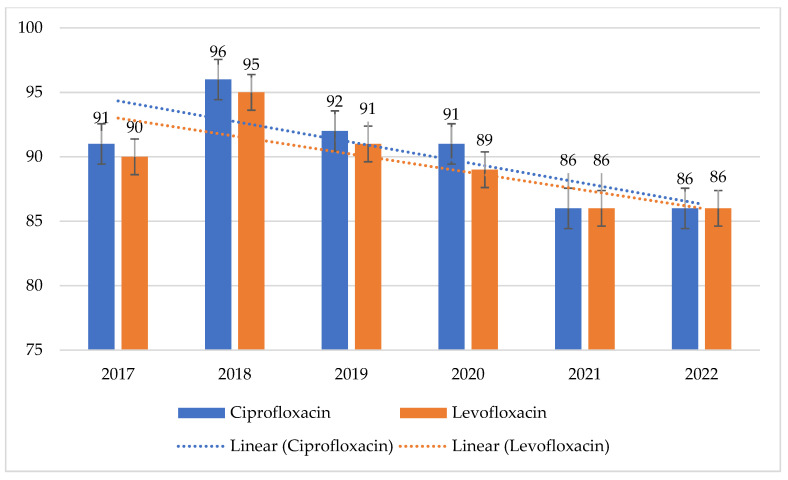
Percentage of MRSA strains resistant to fluoroquinolones.

MRSA strains showed much higher resistance to fluoroquinolones than MSSA strains. For MRSA, this ranged from 86% to 96% (*p* = 0.01661) for ciprofloxacin and from 86% to 95% (*p* = 0.2227) for levofloxacin. At the same time, resistance in MSSA strains ranged from 3% to 9% (*p* = 0.407) for ciprofloxacin and from 3% to 8% (*p* = 0.5715) for levofloxacin. MSSA strains had the highest percentage of resistant strains in 2019, while MRSA strains had the highest percentage of resistant strains in 2018. However, these differences are statistically insignificant (Figure 4 and Figure 5).

**Figure 6:**
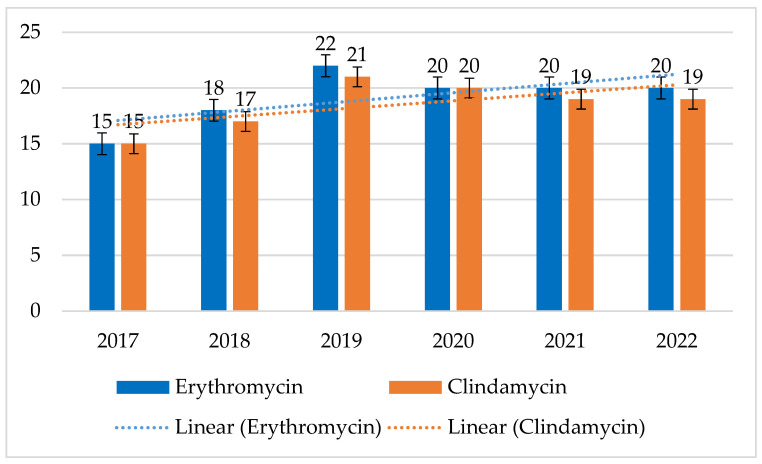
Percentage of MSSA strains resistant to macrolides (erythromycin) and lincosamides (clindamycin).

**Figure 7:**
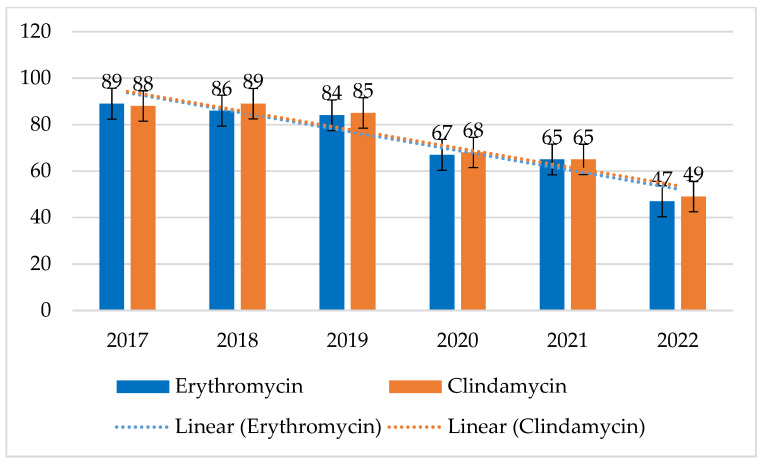
Percentage of MRSA strains resistant to macrolides (erythromycin) and lincosamides (clindamycin).

MRSA strains have shown very high resistance to macrolides and lincosamides ranging from 47% to 89% for both erythromycin and clindamycin, with a continuing downtrend in resistance to both these antibiotics. After 2018, a steady increase in susceptibility to erythromycin can be observed, after 2019 also to clindamycin. In contrast, the percentage of resistant strains among MSSAs was relatively constant and low, ranging from 15% to 22% (*p* = 0.07678) for erythromycin and from 15% to 21% (*p* = 0.1135) for clindamycin (Figure 6 and Figure 7).

**Figure 8:**
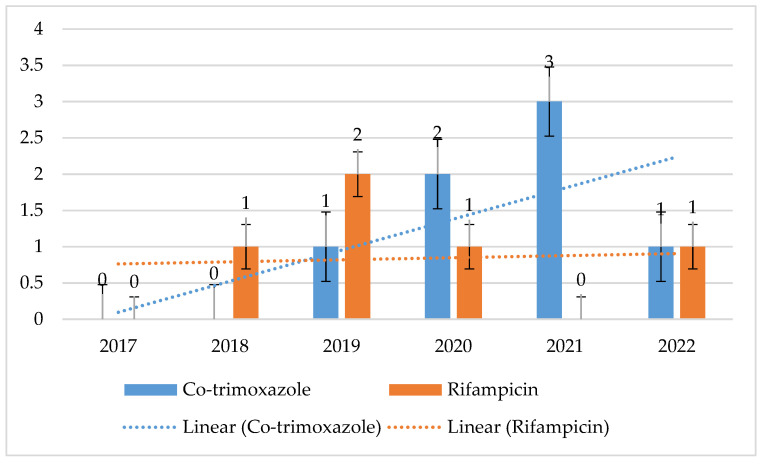
Percentage of MSSA strains resistant to cotrimoxazole and rifampicin.

**Figure 9:**
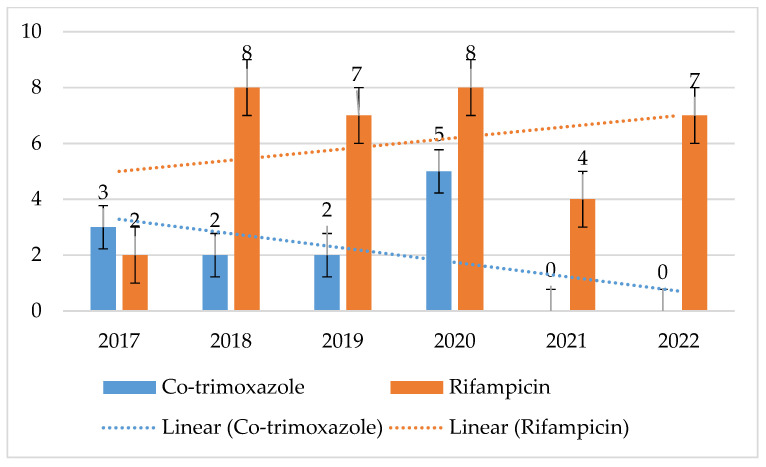
Percentage of MRSA strains resistant to cotrimoxazole and rifampicin.

MRSA and MSSA strains displayed consistent, low resistance to both cotrimoxazole and rifampicin. For MSSA, the maximum percentage of resistant strains was 3% for cotrimoxazole and 2% for rifampicin. In contrast, for MRSA strains, it was up to 5% (*p* = 0.1649) with a clear downward trend for cotrimoxazole, and its maximum value for rifampicin was 8% (*p* = 0.5868) (Figure 8 and Figure 9).

#### 3.1.2. Antibiotic Resistance of *E. faecalis* and *E. faecium* Strains in 2017–2022

For the purposes of analyzing the resistance changes in *E. faecium* and *E. faecalis* strains, antibiotic groups such as fluoroquinolones, glycopeptides, fosfomycin and nitrofurantoin were taken into account. However, among the bacteria isolated from infections in patients of the Multi-Clinic Specialist Hospital in Wroclaw, no strains resistant to linezolid and tigecycline were detected.

**Figure 10:**
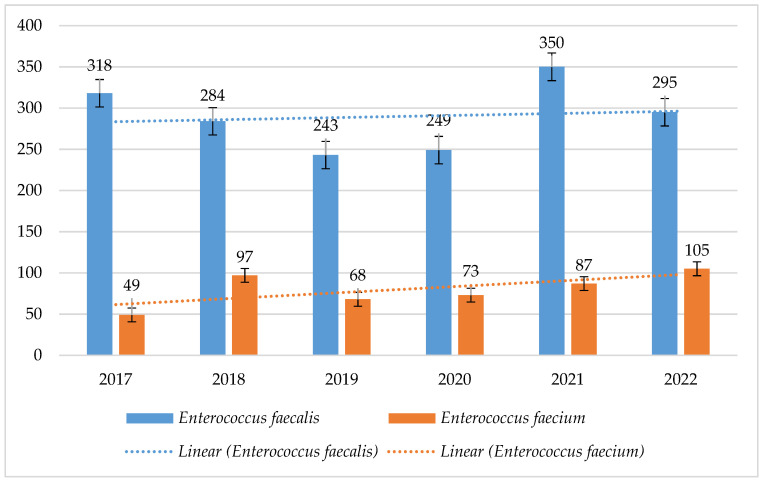
Comparison of the number of *E. faecalis* and *E. faecium* strains between 2017 and 2022.

Over the past 6 years, the number of *E. faecalis* strains was much higher than that of *E. faecium*, ranging from 243 to 350. The highest number of *E. faecalis* was recorded in 2021 (n = 350), while the highest number of *E. faecium* was recorded in 2022 (n = 105) (Figure 10).

**Figure 11:**
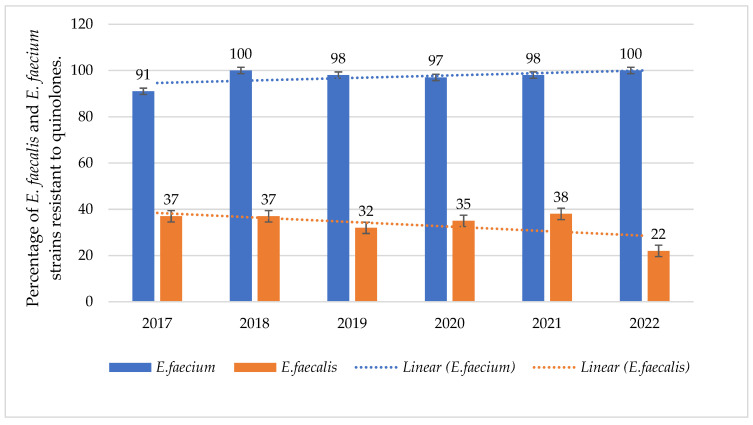
Percentage of *E. faecalis* and *E. faecium* strains resistant to fluoroquinolones.

The resistance of *E. faecalis* to fluoroquinolones was at a constant level and ranged from 22% to 38% (*p* = 0.003578) for levofloxacin, with a decreasing trend. The percentage of *E. faecium* strains resistant to fluoroquinolones was higher, reaching 100% (Figure 11).

In 2018, twice as many *E. faecium* strains were isolated from infections as in 2017 (49 and 97, respectively). The number of VRE strains in hospitals in Poland actually increased in 2018. Moreover, the consumption of cephalosporins II and III gen. in the analyzed hospital increased this year, which could also have contributed to the increase in the number of infections. We also do not exclude the possibility that there was an outbreak in the hospital that was overlooked.

**Figure 12:**
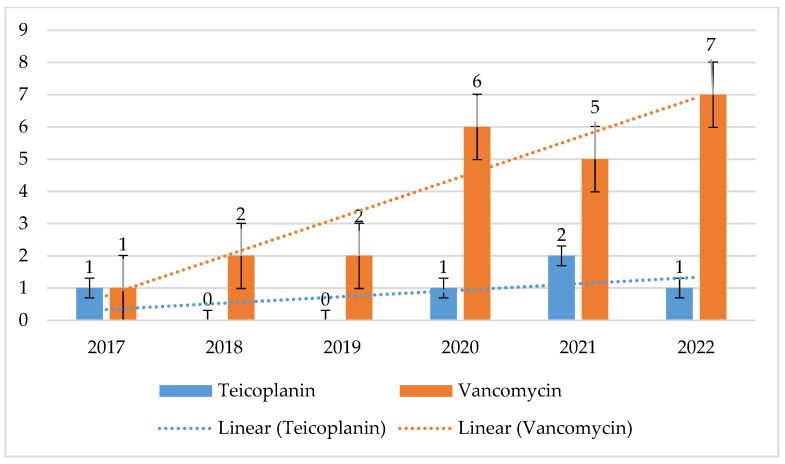
Percentage of glycopeptide-resistant *E. faecalis* strains.

**Figure 13:**
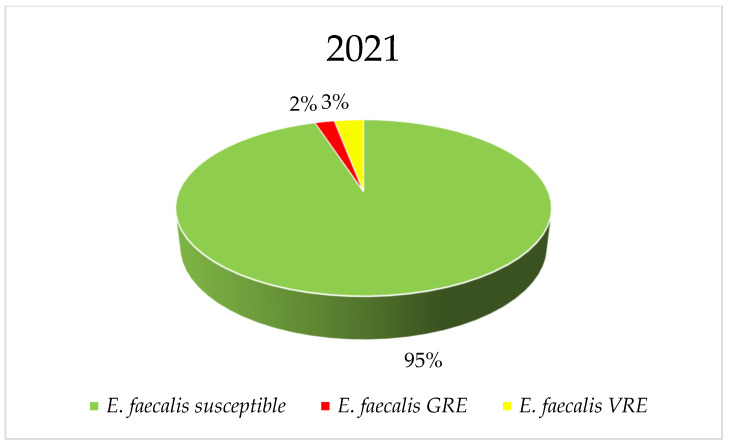

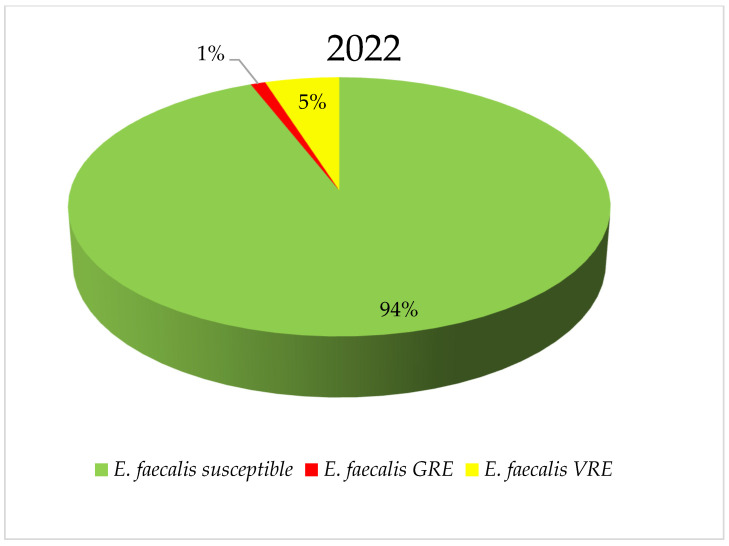
Comparison of the percentage of *E. faecalis* strains sensitive to glycopeptides and GRE and VRE in 2021 and 2022.

**Figure 14:**
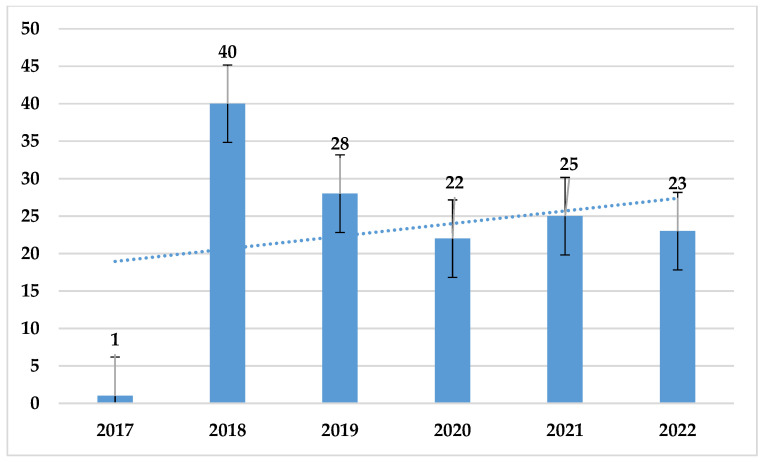
Percentage of *E. faecium* strains resistant to vancomycin.

**Figure 15:**
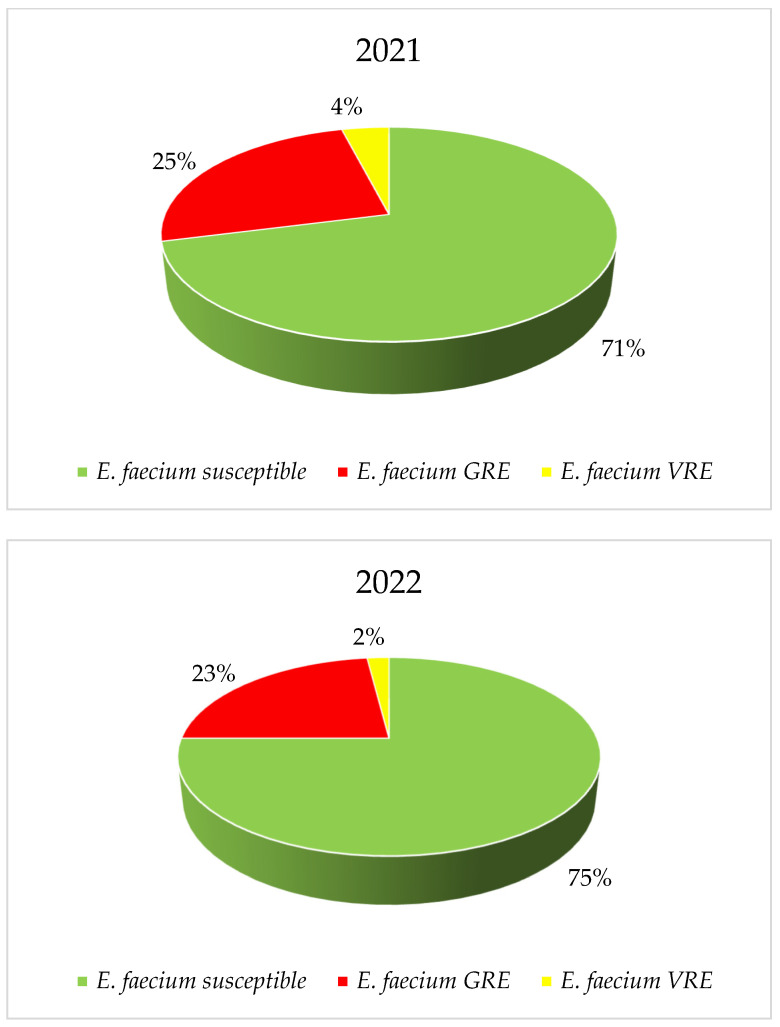
Comparison of the percentage of *E. faecium* strains sensitive to glycopeptides and GRE and VRE in 2021 and 2022.

*E. faecium* strains were characterized by higher vancomycin resistance, reaching 40% (*p* = 0.8323) with a clear downward trend, while the maximum percentage of vancomycin-resistant *E. faecalis* strains was 7% (*p* = 3.154), with a clear upward trend in resistance. Among *E. faecium*, there was a decrease in the percentage of vancomycin-resistant strains over the years. Glycopeptide-sensitive strains predominate for both bacteria in 2021 and 2022. Among the resistant strains in *E. faecalis*, VREs slightly predominate, while GREs (Glycopeptide-Resistant Enterococci) are much more prevalent in *E. faecium* (Figure 12, Figure 13, Figure 14 and Figure 15).

**Figure 16:**
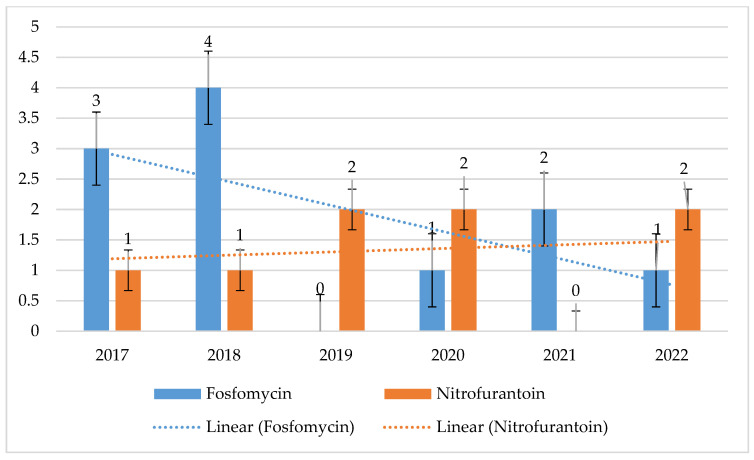
Percentage of *E. faecalis* strains resistant to fosfomycin and nitrofurantoin.

The resistance of *E. faecalis* strains to fosfomycin and nitrofurantoin was at a low level and ranged from 0% to 4% (*p* = 0.01966) and 0% to 2% (*p* = 0.7662), respectively. Resistance to these antibiotics was not analyzed among *E. faecium* strains (Figure 16). As with *E. faecium*, no *E. faecalis* strain resistant to linezolid was recorded over the analyzed 6-year period.

#### 3.1.3. Antibiotic Resistance of *S. pneumoniae* Strains in 2017–2022

In order to observe changes in the resistance of *S. pneumoniae*, the sensitivity of these bacteria to antibiotic groups such as penicillins, cephalosporins, macrolides, lincosamides, tetracyclines and cotrimoxazole was analyzed. There was no resistance to glycopeptides and linezolid among the *S. pneumoniae* isolated from infections in the selected hospital.

The highest number of *S. pneumoniae* strains was isolated in 2018, while the lowest was in 2021.

**Figure 17:**
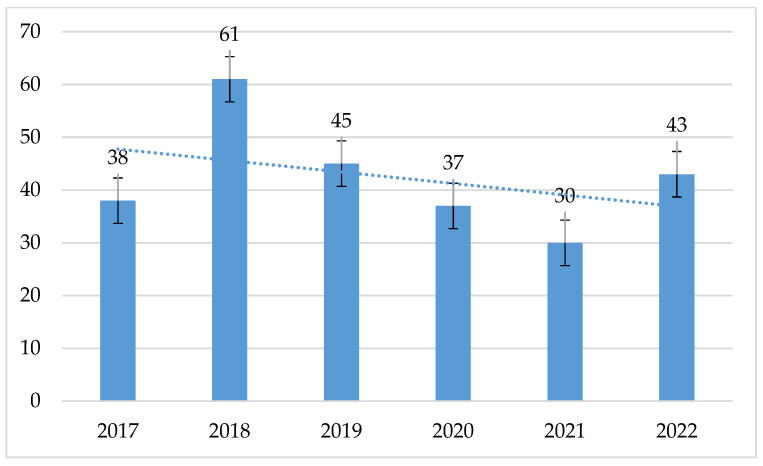
Number of *S. pneumoniae* strains isolated in 2017–2022.

**Figure 18:**
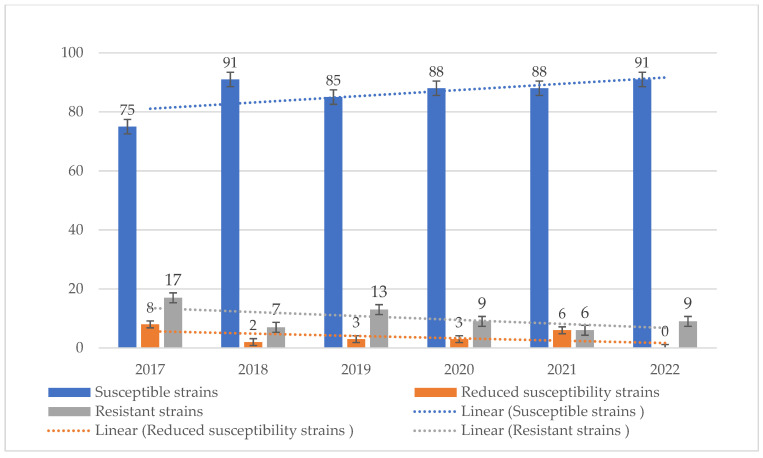
Comparison of the percentage of *S. pneumoniae* strains that are susceptible, susceptible with increased exposure and resistant to ampicillin.

**Figure 19:**
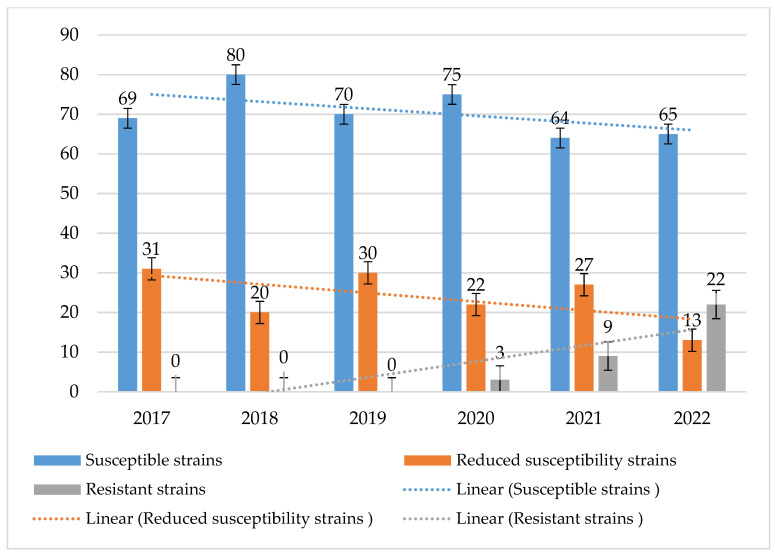
Comparison of the percentage of *S. pneumoniae* strains susceptible, susceptible with increased exposure and resistant to benzylpenicillin.

Susceptible strains dominated in the case of ampicillin, with percentages ranging from 75% to 91%. In addition, each year, a higher percentage of resistant strains was observed than of those with reduced susceptibility (susceptible with increased exposure to the drug) and ranged from 6% to 17% (Figure 17 and Figure 18). Taking into account the resistance of *S. pneumoniae* to benzylpenicillin, it can be said that in this case sensitive strains also prevailed. Their percentage ranged from 64% to 80%. In 2017–2019, almost no so-called highly resistant strains to this antibiotic were observed. Over the past 2 years, there has been an increase in the resistance of *S. pneumoniae* to benzylpenicillin. The percentage of strains with reduced sensitivity has also remained stable (much higher than for ampicillin) and ranges from 20% to 31%. In the period 2021–2022, there was a significant increase in the number of increased exposure and resistant strains (Figure 19).

**Figure 20:**
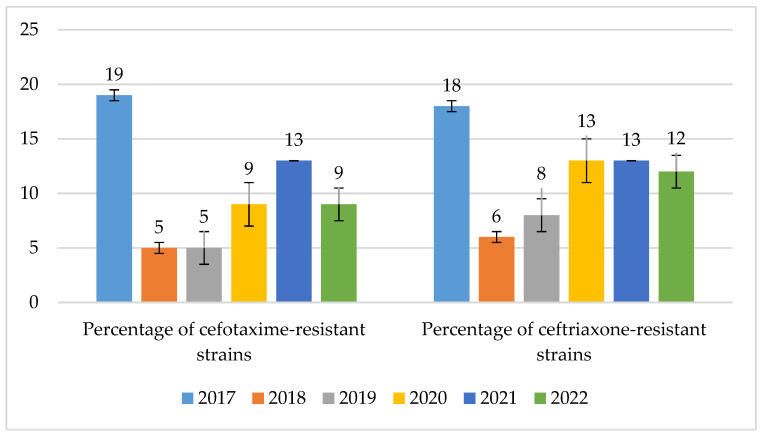
Percentage of *S. pneumoniae* strains resistant to cephalosporins.

The percentage of cephalosporin-resistant strains was at a low level, ranging from 5% to 19% (*p* = 0.79) for cefotaxime and 6% to 18% (*p* = 0.985) for ceftriaxone. In contrast, the highest percentage of resistant strains occurred in 2017 (Figure 20).

**Figure 21:**
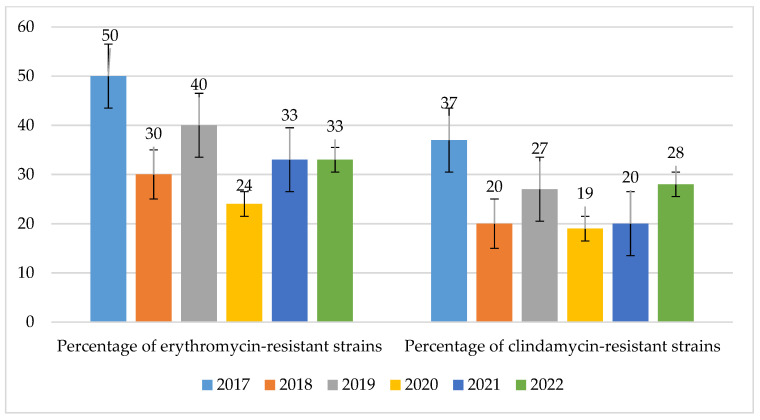
Percentage of *S. pneumoniae* strains resistant to macrolides (erythromycin) and lincosamides (clindamycin).

The resistance of *S. pneumoniae* strains to macrolides was at an average level and ranged from 24% in 2020 to 50% in 2017 (*p* = 0.2168). The percentage of strains resistant to lincosamides was at a similar level and ranged from 19% in 2020 to 37% in 2017 (*p* = 0.5431) (Figure 21).

**Figure 22:**
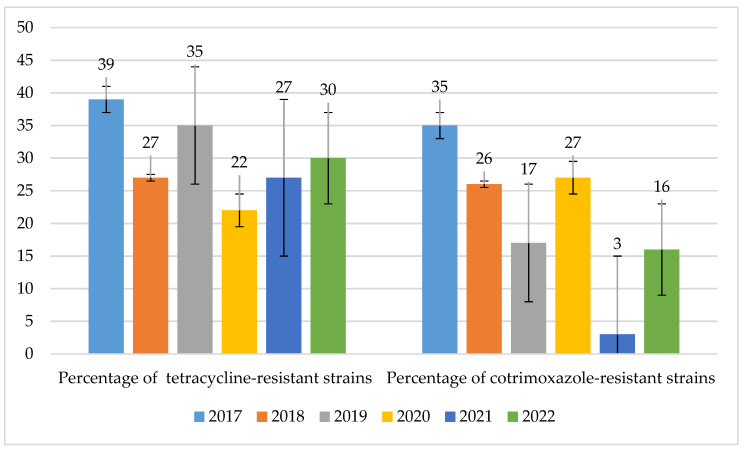
Percentage of *S. pneumoniae* strains resistant to tetracyclines and cotrimoxazole.

The percentages of strains resistant to tetracyclines and cotrimoxazole were at similar levels, ranging from 22% to 39% (*p* = 0.435) and 17% to 35% (*p* = 0.01084), respectively (in 2017–2020). However, in the case of cotrimoxazole, a significant decrease in the percentage of resistant strains to 3% was observed in 2021 (Figure 22).

#### 3.1.4. Statistical Analysis Results

The pattern of drug resistance differed among the studied microorganisms as well as among the studied drugs (Table 3). Among the thirty-five strain x drug combinations, fourteen showed a statistically significant (*p* < 0.05) or marginally statistically significant trend of increase (six strains) or decrease (eight strains).

### 3.2. Consumption of Antibiotics in Hospitals in the Analyzed Period 2017–2022

**Figure 23:**
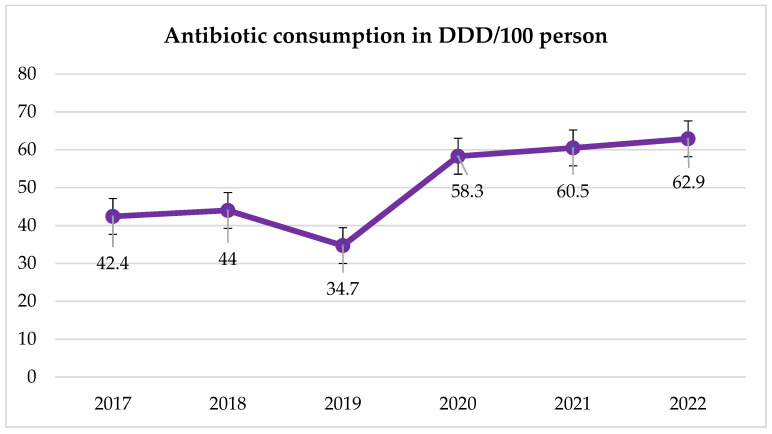
Trend in the consumption of all antibiotic groups during the period under study.

In the analyzed six-year period, from 2020 (beginning of the pandemic), there was a sharp increase in the use of antibiotics in hospitals, which continued in the following years with a slight upward trend (Figure 23).

**Figure 24:**
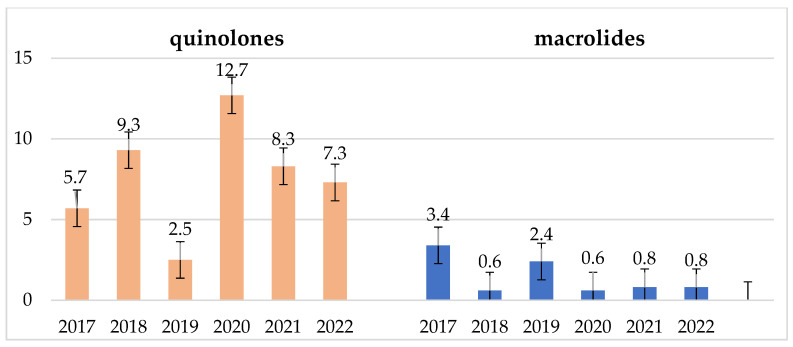
Consumption of fluoroquinolones and macrolides in the examined 6-year period in DDD/100 persons.

In some groups of antibiotics, a significant increase in consumption in DDD/100 person-days was recorded. This is most visible in the case of fluoroquinolones (Table 4, Figure 24). The overuse of these antibiotics during the pandemic and in subsequent years is visible in the trends of high resistance to these antibiotics in MRSA strains and *E. faecium* and *E. faecalis* (Figure 11 and Figure 12). *S. pneumoniae* strains also show high resistance to levofloxacin (resistance to levofloxacin increased to 100% in 2021 and 2022). No such trend was observed in the case of macrolides—their consumption remains at a constant, quite low level (Figure 22).

**Figure 25:**
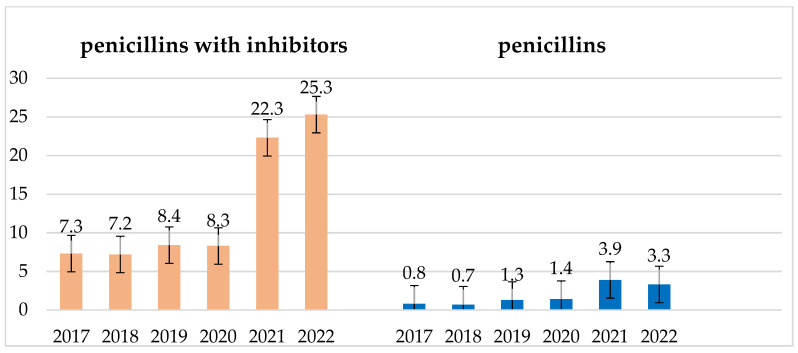
Consumption of penicillins and penicillins with inhibitors in the examined 6-year period in DDD/100 persons.

**Figure 26:**
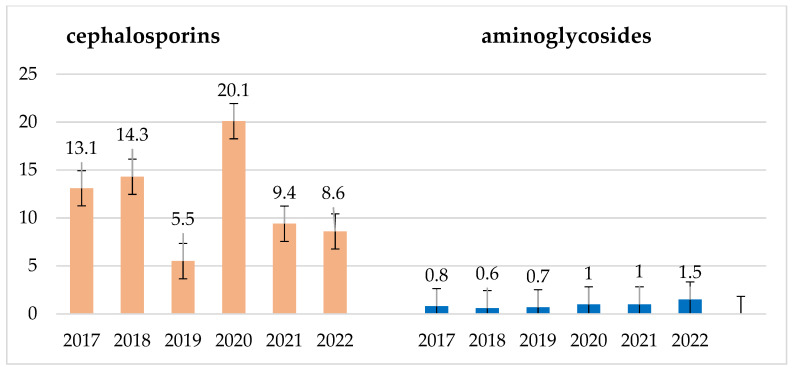
Consumption of cephalosporins and aminoglycosides in the studied 6-year period in DDD/100 persons.

In the years 2020–2022, there was an increase in the consumption of cephalosporins, and in the years 2021–2022, there was an increase in the consumption of penicillins and penicillins with inhibitors (Figure 25). This drove an increase in the number of infections caused by *Enterococcus* strains as well as the emergence of more penicillin-resistant strains of the species *E. faecium* and *S. pneumoniae*. However, the consumption of aminoglycosides in the examined period was quite low and practically constant (Figure 26).

## 4. Discussion

Recently, antimicrobial therapy is becoming much more complicated, and there are situations in which microorganisms are resistant to last-chance antibiotics [17,18,19].

The most common bacterial carriers of resistance genes that are of great clinical importance in the group of Gram-positive cocci are primarily *S. aureus, Enterococcus* spp. and *S. pneumoniae* [20]. A significant portion of the microorganisms analyzed in this study include those that are on the list of “priority pathogens” published since 2017 and constantly updated by the WHO [21]. Therefore, these bacteria and changes in their resistance over the years 2017–2022 were analyzed in this paper.

Currently, apart from the production of carbapenemases by intestinal bacteria and non-fermenting bacilli [22,23], the biggest problem in the hospital environment is the resistance of *S. aureus* to methicillin or the resistance of enterococci to vancomycin [9]. As MDR strains, they may be markers of multidrug resistance [24]. Gram-positive bacteria classified as alarm pathogens include *S. aureus* resistant to methicillin (MRSA), glycopeptides (VRSA/VISA) or oxazolidinones and *Enterococcus* spp. resistant to glycopeptides (VREs) or oxazolidinones, as well as *S. pneumoniae* resistant to third-generation cephalosporins or penicillin [25]. Epidemiological data from the United States indicate that MRSA infections are one of the leading causes of mortality among patients [26,27].

In many countries, a decrease in the number of infections caused by *S. aureus* has been observed in recent years [28]. The Central Asian and European Surveillance of Antimicrobial Resistance Network (CAESAR) covers the surveillance of antimicrobial resistance (AMR) for all countries in the WHO European Region that are not covered by the European Antimicrobial Resistance Surveillance Network (EARS-Net). Taking into account the CAESAR network data, *S. aureus* accounted for 17% of isolates in 2020 [29]. In the analyzed hospital, there was also a clear drop in the number of isolated *S. aureus* strains, and their average percentage share in the annual pool of infections was 20%—only slightly higher than the average for the countries of Europe and Central and Eastern Asia. In Europe, a downward trend in the number of MRSA strains has been observed in recent years [29,30,31]. Since 2015, a statistically significant decrease in the percentage of *S. aureus* resistant to methicillin has been observed in Europe from 19% to approximately 15.5% [20]. This was also observed in the analyzed hospital, where the number of methicillin-resistant *S. aureus* strains decreased significantly after 2018. However, it should be noted that the overall ratio of the number of MRSA and MSSA strains in recent years has been relatively stable in the analyzed hospital. Based on the results obtained during a five-year analysis conducted in hospitals in Macau, the most densely populated region in the world, a significant increase in the detection rate of MRSA strains was observed, which was 30.0% in 2017 and increased to as much as 47.9% in 2022 [32]. Asia dominates the world in terms of the share of MRSA strains in human infections, which reached as much as 60% in Yunnan Province, China [33]. Significant differences in the share of MRSA in infections were noted in African countries, where these indications ranged from 1% to 84.1% [34]. In turn, epidemiological data from the Gulf Cooperation Council countries covering the period 2005–2019 indicate a more than four-fold increase in the detection of these pathogens from infections, which ranged from 9% to 38% [35]. In the case of Japan and Taiwan, where preventive infection control practices for MRSA etiology were introduced, a decrease in the share of these microorganisms in infections was observed [36].

In Europe, up to 17% of isolated bacteria are resistant to methicillin, while in Poland, this percentage is similar and stands at approximately 16% [29,30,37]. Based on data from the analyzed hospital, it appears that in recent years, MRSA strains were the etiological factor of 18–28% of *S. aureus* infections. This percentage is therefore slightly higher than the EU/EEA average but has been declining in recent years. The higher number of *S. aureus* infections in the years 2017–2019 may be due to the fact that not all surgical wards had procedures for detecting and eradicating these microorganisms. In the following years, greater monitoring of compliance with these procedures was applied. The MRSA strains isolated in the analyzed hospital show significant sensitivity to antibiotic groups other than β-lactams, such as aminoglycosides, tetracyclines, cotrimoxazole, linezolid and vancomycin.

Gentamicin resistance was found in a small percentage of *S. aureus* strains isolated from nosocomial infections in the analyzed hospital. The share of resistant strains reached a maximum of 4% for MSSA and 10% for MRSA. As the EARS-Net data show, this resistance rate was also lower than the Polish average of 4% [29,30,37]. The analysis of the consumption of aminoglycosides in the analyzed period showed a very sparing and rational use of these antibiotics, remaining at a constant, low level. This certainly resulted in a very low level of resistance to gentamicin in both MSSA and MRSA strains.

The resistance of these bacteria to cotrimoxazole and rifampicin was also low and amounted to a maximum of 3% and 2% for MSSA strains and 5% and 8% for MRSA. The use of these antibiotics in the hospital also remains at a very low, relatively constant level.

As a representative of fluoroquinolones, ciprofloxacin is the antibiotic to which *S. aureus* displays the second-highest resistance, after methicillin. According to EARS-Net data, in Poland, a lack of sensitivity to this group of drugs was found on average in 16% of strains [29,30,37]. Among MSSA isolates from the Specialist Hospital in Wrocław, the maximum percentage of resistant strains was 9%, which is lower than the Polish average (data from 2019). In the case of MRSA strains, almost 100% lack of sensitivity to fluoroquinolones was found, which was certainly related to the excessive consumption of these drugs, especially during the pandemic. The increased use of antibiotics during the pandemic resulted from lack of knowledge and fear related to the bacterial complications of SARS-CoV-2 infection. In severe cases of infection or when CRP increased, broad-spectrum antibiotics were routinely used in over 70% of patients. Current research suggests that only 8–14% of COVID-19 patients have bacterial co-infection. Moreover, the increase in CRP observed with the severity of the disease process is not related to bacterial co-infection but to the action of interleukin-6, released in response to SARS-CoV-2 infection, stimulating the production of CRP. The excessive use of antibiotics always leads to increased bacterial resistance.

Currently, the percentage of fluoroquinolone resistance shows a decreasing tendency, which may be related to the recommendations issued by the hospital at the end of 2020 to avoid the use of fluoroquinolones, especially in empirical therapy. In 2017–2018, MRSA strains showed almost 90% lack of sensitivity to erythromycin and clindamycin (macrolides and lincosamides), despite the fact that these antibiotics were not overused in therapy, while in recent years, the share of resistant strains in infections has shown a clear downward trend. In turn, the resistance of MSSA strains to these antibiotics was quite low but constant, ranging from 15% to 22% and 15% to 21%, respectively.

Streptococci of the genus *Enterococcus* are characterized by quite broad natural resistance, which includes cephalosporins, trimethoprim/sulfamethoxazole, lincosamides and low concentrations of aminoglycosides [38]. Additionally, *E. faecium* is characterized by a lack of sensitivity to penicillins, while *E. faecalis* retains this sensitivity [39]. *E. faecalis*, on the other hand, has a naturally reduced sensitivity to lincosamides and streptogramins [40]. In addition to innate resistance, there may also be acquired resistance, which is related to a mutation in the bacterial genetic material or results from the transfer of a given trait using mobile genetic elements [38]. This mechanism leads to the development of multidrug-resistant strains, which are therefore very difficult to combat. These bacteria may acquire resistance to β-lactams, glycopeptides, high concentrations of aminoglycosides, tetracyclines, macrolides, linezolid or fluoroquinolones, but of all the developed resistance mechanisms, the most important are HLAR, VRE and LRE [39,41]. SENTRY, as one of the antimicrobial surveillance programs, monitors worldwide changes in the resistance patterns of key pathogens. One study analyzed increasing drug resistance trends for almost 50,000 clinical isolates of enterococci from different geographical regions (Asia, Europe, South and North America) that were isolated from infections between 1997 and 2016 [42]. In CAESAR network countries, an increased incidence of infections caused by *Enterococcus* spp. bacteria has recently been observed [31]. A similar situation also occurred in the analyzed hospital, where in the case of infections caused by *E. faecium*, a constant increase in their number was observed, while the number of *E. faecalis* isolates reached the highest value over the last few years in 2021.

In the years 2007–2015, in the European Union, the average number of infections and deaths related to vancomycin-resistant strains of the genus *Enterococcus* spp. almost doubled. The increase in the share of strains with the VRE phenotype (recorded since 2016) contributed to a further increase in the number of infections of this etiology [43]. The VRE phenotype was most common in North America (21.6%), while a characteristic feature of all *Enterococcus* spp. strains tested in the SENTRY program, regardless of the region, was a steady decrease in susceptibility to ampicillin and vancomycin [43]. In Poland, the average percentage of *E. faecalis* and *E. faecium* strains insensitive to vancomycin was 3% and 37%, respectively, which is one of the highest figures in Europe [29,30,37]. Moreover, in the case of *E. faecium*, an increase in the number of VRE strains was observed year after year [31]. The increase in the number of infections caused by strains of the *Enterococcus* genus, also observed in the analyzed hospital, was most likely related to the overuse of cephalosporins during the pandemic, which are the antibiotics that generate the selection of these bacteria due to their natural resistance to this drug group. In turn, the percentage of vancomycin-resistant *E. faecalis* strains in the Specialist Hospital was low, reaching a maximum of 7%. For *E. faecium* strains, this share reached as much as 41% in 2018, which corresponds to the average for Poland in the analyzed period. What makes us optimistic is the fact that in recent years there has been a downward trend noted. In China, however, the percentage of *E. faecalis* and *E. faecium* VRE strains was very low and amounted to <1% and <5%, respectively [44]. This was probably owing to the rare use of these preparations due to the lack of access to the oral form [44].

It is also worth noting that VRE strains predominated among *E. faecalis* strains insensitive to glycopeptides isolated from nosocomial infections at the Specialist Hospital in Wrocław. In the case of *E. faecium*, GRE strains predominated, i.e., strains resistant to all glycopeptides, which resulted in the exclusion of this entire group of drugs from therapeutic options. Since 2019, an increase in the number of strains sensitive to glycopeptides has been observed in the analyzed hospital, and in 2022, the share of *E. faecium* with the GRE phenotype was 23%. In the case of *E. faecalis,* VRE strains often remain sensitive to fosfomycin and nitrofurantoin but show quite high (22–43%) resistance to levofloxacin. In turn, *E. faecium* isolates with this resistance phenotype exhibit almost 100% resistance to levofloxacin. This is certainly related, as in the case of *S. aureus*, to the overuse of fluoroquinolones in the hospital. We hope that the visible downward trend in recent years is related to the recommendations of the Nosocomial Infection Control Team not to use fluoroquinolones in empirical therapy (also due to the recently described serious side effects).

In recent years, *S. pneumoniae* cocci have also developed many resistance mechanisms, mainly to penicillin but also to antibiotics from other groups. In addition to resistance to penicillins, resistance to other β-lactams, macrolides, lincosamides, sulfonamides and fluoroquinolones is also observed [45]. Since 2015, a statistically significant decrease in the percentage of *S. pneumoniae* resistant to penicillin has been observed in Europe from 14.2% to 12.1% [20]. According to data from the CAESAR network, the number of *S. pneumoniae* isolates decreased in 2019–2021, which is also reflected in the data from the Hygiene and Epidemiology Department from the analyzed hospital, while in 2022, according to the data obtained, a slight increase in infections with this etiology was noted [31]. Materials collected in the CHINET network show that in China, the increasing resistance of *S. pneumoniae* strains to penicillins in the years 2015–2017, ranging from 10.5% to 13.5%, raises much concern [44]. Data from the United States indicate that in the case of *S. pneumoniae* strains isolated from infections, the share of resistance to β-lactam antibiotics was the highest compared to resistance to macrolides or fluoroquinolones [46].

On the other hand, after 2019, in Europe, a stabilization of the share of resistant strains was observed at a level not significantly different from 12% [30]. In the case of Poland, we are dealing with a slightly higher percentage of non-susceptible strains, ranging from 15.5% to 16.6% in 2017–2019 [30,37,47]. It is worth noting, however, that among non-susceptible strains, completely resistant strains constitute on average only about 4% [30,37,47]. The data from the analyzed hospital show that the percentage of strains insensitive to ampicillin ranged from 6% to 17%, which corresponded to the national average. In 2017, the percentage of strains resistant to this antibiotic reached 17%, while in 2022, their share decreased to 9%, i.e., below the national average.

In turn, in the case of benzylpenicillin, based on the data obtained from the examined hospital, it was found that the percentage of non-susceptible strains has been systematically increasing since 2020, reaching 22% in 2022. Strains with reduced sensitivity predominated, and until 2020, there were practically no resistant strains. Recently, there has been a significant increase in the share of strains resistant to benzylpenicillin, which clearly coincides with the increase in the use of penicillins and penicillins with inhibitors in the hospital in recent years. However, most of these strains remain sensitive to cephalosporins. Among the *S. pneumoniae* strains isolated in the analyzed hospital, resistance to third-generation cephalosporins ranged quite widely, from 5% to 19%. According to the EARS-Net, *S. pneumoniae* bacteria showed higher sensitivity to ceftriaxone. However, such conclusions cannot be drawn on the basis of this study [29,30,31]. The data from the analyzed facility indicate that bacterial resistance to cefotaxime and ceftriaxone was at a very similar level. The constant increase in the resistance of these bacteria to this group of antibiotics observed in recent years coincided with the rapid growth in the consumption of the drugs in question, which took place in 2020–2021. In the same period, the data obtained for the entire country looked slightly better, and the percentage of non-susceptible strains was on average 5.5% [30,37]. In the Specialist Hospital, this percentage approached the national average only in 2018–2019. This shows how dangerous the overuse of β-lactam antibiotics, especially penicillins with inhibitors and cephalosporins, may be for the increase in strain resistance. This is especially visible in the increase in the number of infections caused by strains of the *Enterococcus* genus, *E. faecium* (naturally resistant to penicillins), as well as by *S. pneumoniae* strains insensitive to penicillins. This is also of great importance for the increase in the resistance of *Enterobacterales* bacilli (increase in the number of ESBL+ strains), which was proven in the authors’ previous study on the analyzed hospital [22]. Macrolides are also a group of antibiotics to which pneumonia bacteria have developed the highest resistance. Based on data from the CARSS network, it is estimated that up to 90% of *S. pneumoniae* strains occurring in China may be resistant to erythromycin [34]. The data provided by the EARS-Net look slightly more optimistic. According to these data, the percentage of resistant strains in Poland is on average 25% [19,20,28,29,30,31]. In the analyzed Specialist Hospital, this resistance is much higher, and only in 2020 it came close to the national average. In 2021–2022, the percentage of strains resistant to erythromycin was as high as 33%. Based on the analysis of data conducted by multicenter programs monitoring the drug resistance of bacterial pathogens, it was found that in the case of *S. pneumoniae*, resistance to macrolides varied regionally, while fluoroquinolones are still an available therapeutic option in infections caused by this etiological agent [48].

This resistance is related to the MLS mechanism, which usually also causes cross-resistance to clindamycin. An increase in *S. pneumoniae* resistance to 100% was observed in 2017 and 2019, where the use of macrolides quadrupled. These bacteria also showed resistance to tetracyclines and cotrimoxazole, which has remained in the range of 22–39% and 3–35% in recent years. Interestingly, in 2017 and 2019, the highest consumption of tetracyclines in hospitals was observed, which coincided with the highest resistance of *S. pneumoniae* to this group of antibiotics.

The analysis of strains isolated from infections in the Specialist Hospital allowed us to trace the development of drug resistance in the group of Gram-positive cocci, as the most common etiological factors of nosocomial infections. Moreover, it made it possible to compare the obtained data with antibiotic consumption reports. A correlation was observed between the increase in the resistance of selected microorganisms isolated from hospital infections and the increase in the use of antibiotics. A disturbing phenomenon observed in this study was the appearance of an increased number of VREs.

The trends observed in the studies regarding the increasing resistance of Gram+ pathogens mobilized the hospital to take specific actions. One of them is monitoring the procedures for detecting and eradicating *S. aureus* carriage in surgical wards. Another action is to update the perioperative prevention procedure and the empirical therapy procedure in the hospital. For example, fluoroquinolones were almost completely withdrawn from empirical therapy (also due to serious side effects documented in recent years). These analyses need to be continued to track the development of resistance over a longer period of time. This applies especially to the years following the COVID-19 pandemic, during which an irrational increase in the use of antibiotics contributed to a decrease in the sensitivity of many of the tested pathogens.

The presented retrospective analysis of the resistance of Gram+ pathogens is the result of reports prepared for this hospital, presented and discussed at meetings with doctors. The resistance mechanisms of microorganisms isolated in the hospital are monitored, and such reports are made every year. They are placed in a computer database with access given to all users. This allows for the tracking of new resistance mechanisms and the correction of therapeutic procedures in the hospital.

This study is only a retrospective analysis of the results of microbiological tests in patients hospitalized in the presented hospital in 2017–2022. The authors are aware of the significant limitations of the presented data. For the purposes of the hospital, resistance trends in individual hospital departments were also analyzed. Resistance analyses of strains isolated from specific clinical samples were also performed. This was useful for doctors in the hospital, but such an analysis in one paper would be complicated and unclear. For this reason, the retrospective analysis of microbial resistance is presented in three parts: *Enterobacterales* bacilli—*E. coli* and *Klebsiella*; non-fermentative bacilli—*Acinetobacter* and *Pseudomonas*; and Gram+ cocci. The two previous parts were published in this journal in the year 2023. The current work is the final part of the overall analysis. It seems to us that despite the many limitations of such studies, we managed to capture certain trends in increasing resistance resulting, for example, from the consumption of antibiotics. The analysis has already resulted in many changes to hospital procedures and formularies. It seems that, regardless of possible errors, such monitoring will also allow for the tracking of the epidemiological situation in a multiprofile hospital in the future.

## 5. Conclusions

In the years 2020–2023, a significant increase in the consumption of antibiotics, in particular fluoroquinolones, was observed in the analyzed multiprofile hospital. Gram-positive pathogen strains of MRSA, *E. faecalis*, *E. faecium* and *S. pneumoniae* have proven to be highly resistant to these antibiotics. Regardless of certain limitations, one of the practical effects of this research will be the ability to determine changes in the drug resistance of the analyzed microorganisms and detect new trends in resistance mechanisms. The analysis of the obtained results indicates the need for the constant monitoring of bacterial resistance patterns to available antibacterial drugs, especially in the case of priority pathogens that constitute a global problem. Choosing the appropriate treatment may be a factor limiting the spread of these microorganisms, which is crucial in preventing the infections they cause.

## Figures and Tables

**Table 1:** Number of tested strains of individual Gram-positive microorganisms isolated from hospital infections in 2017–2022.

Year/Strain	2017 (%)	2018 (%)	2019 (%)	2020 (%)	2021 (%)	2022 (%)
*S. aureus*	532 (56.8)	627 (58.7)	445 (55.6)	365 (50.4)	348 (42.7)	361 (44.9)
*E. faecalis*	318 (33.9)	284 (26.6)	243 (30.3)	249 (34.4)	350 (42.9)	295 (36.7)
*E. faecium*	49 (5.2)	97 (9.1)	68 (8.5)	73 (10.1)	87 (10.7)	105 (13.1)
*S. pneumoniae*	38 (4.1)	61 (5.7)	45 (5.6)	37 (5.1)	30 (3.7)	43 (5.3)
Total	937	1069	801	724	815	804

**Table 2:** Gram-positive bacterial species and antibiotics selected for resistance analysis.

Microorganism	Selected Antibiotics
*Staphylococcus aureus* MS/MR *	Gentamicin, amikacin, erythromycin, clindamycin, levofloxacin, ciprofloxacin, rifampicin and cotrimoxazole
*Enterococcus faecalis*	Levofloxacin, teicoplanin, vancomycin, nitrofurantoin, fosfomycin and linezolid
*Enterococcus faecium*	Levofloxacin, teicoplanin, vancomycin and linezolid
*Streptococcus pneumoniae*	Cefotaxime, ceftriaxone, erythromycin, clindamycin, benzylpenicillin, ampicillin, tetracycline and cotrimoxazole

* *Staphylococcus aureus* MS/MR—*Staphylococcus aureus* methicillin-susceptible/methicillin-resistant.

**Table 3:** Trend analysis of the resistance of the studied microorganisms to the applied drugs using the Cochran–Armitage test. The table presents the *p*-values in the Cochran–Armitage test, and arrows indicate the trend direction. Question marks denote *p* < 0.1.

Drug	*S. aureus* MSSA		*S. aureus* MRSA		*S. pneumoniae* (Resistant Strains)		*S. pneumoniae* (Reduced Susceptibility Strains)		*E. faecalis*		*E. faecium*	
Amikacin	0.1657		0.1815									
Gentamicin	0.0103	↓	0.0008	↑								
Ciprofloxacin	0.4070		0.0166	↓					0.0755	↓	0.3026	
Levofloxacin	0.5715		0.2227						0.0036	↓	0.0776	↑
Erythromycin	0.0765	↑	<0.0001	↓	0.2168							
Clindamycin	0.1135		<0.0001	↓	0.5431							
Tetracycline					0.4350							
Cotrimoxazole	0.0006	↑	0.1649		0.0108	↓						
Rifampicin	0.6470		0.5868									
Cefotaxime					0.7900							
Ceftriaxone					0.9850							
Ampicillin					0.4349		0.2849					
Benzylpenicillin					<0.0001	↑	0.4881					
Teicoplanin									0.1012			
Vancomycin									<0.0001	↑	0.8323	
Fosfomycin									0.0197	↓		
Nitrofurantoin									0.7662			

**Table 4:** Consumption over six years in selected key groups of antibiotics.

	Antibiotic Consumption [DDD/100 Person-Days]												
Year	TETs	PENs	PENs + ins.	C II	C III	C IV	CARs	MACs	LINs	AMs	CHs	GPs	Total
2017	1.0	0.8	7.3	9.3	2.6	0.1	0.9	3.4	0.2	0.8	5.7	0.6	42.4
2018	0.6	0.7	7.2	11.0	3.1	0.2	1.2	0.6	0.6	0.6	9.3	0.9	44.0
2019	2.4	1.3	8.4	1.4	2.7	0.2	2.4	2.4	0.8	0.7	2.5	1.3	34.7
2020	0.8	1.4	8.3	2.6	15.7	0.3	2.0	0.6	1.1	1.0	12.7	1.1	58.3
2021	0.7	3.9	22.3	1.8	7.4	0.2	2.1	0.8	1.0	1.0	8.3	1.4	60.5
2022	0.7	3.3	25.3	1.8	4.7	0.2	2.8	0.8	1.2	1.5	7.3	1.4	62.9

TETs—tetracyclines; PENs—penicillins; PENs + ins—penicillins with inhibitors (amoxicillin/clavulanic acid, piperacillin/tazobactam); C II—2nd-generation cephalosporins (cefuroxime); C III—3rd-generation cephalosporins (cefotaxime, ceftriaxone, ceftazidime); C IV—4th-generation cephalosporins (cefepime); CARs—carbapenems (imipenem, meropenem); MACs—macrolides (clarythromycin, azithromycin); LINs—lincosamides (clindamycin); AMs—aminoglycosides (amikacin, gentamycin); CHs—fluoroquinolones (ciprofloxacin, levofloxacin); GPs—glycopeptides (vancomycin, teicoplanin). Total—includes all antibiotics, including selected ones. Clear increasing trends that may contribute to the increase in drug resistance of strains are marked in red.

## Data Availability

Data are contained within the article.
